# The impact of frontal lesions after mild to moderate traumatic brain injury on frontal network measures

**DOI:** 10.1371/journal.pone.0287832

**Published:** 2023-11-30

**Authors:** Sandra E. Rakers, Edith J. Liemburg, Harm J. van der Horn, Jan Cees de Groot, Jacoba M. Spikman, Joukje van der Naalt

**Affiliations:** 1 Department of Clinical Neuropsychology, University Medical Center Groningen, University of Groningen, Groningen, The Netherlands; 2 BCN Neuroimaging Center of the Department of Neuroscience, University Medical Center Groningen, University of Groningen, Groningen, The Netherlands; 3 Department of Neurology, University Medical Center Groningen, University of Groningen, Groningen, the Netherlands; 4 Department of Radiology, Medical Imaging Centre, Groningen, University Medical Center Groningen, University of Groningen, Groningen, the Netherlands; University at Buffalo, UNITED STATES

## Abstract

To investigate the impact of frontal macro-structural lesions on intrinsic network measures, we examined brain network function during resting-state fMRI in patients with frontal lesions in the subacute phase after mild to moderate traumatic brain injury. Additionally, network function was related to neuropsychological performances. 17 patients with frontal lesions, identified on admission CT after mild to moderate trauma, were compared to 30 traumatic brain injury patients without frontal lesions and 20 healthy controls. Three months post-injury, we acquired fMRI scans and neuropsychological assessments (measuring frontal executive functions and information processing speed). Using independent component analysis, the activity of and connectivity between network components (largely located in the prefrontal cortex) and relations with neuropsychological measures were examined and compared across groups. The analysis yielded five predominantly frontal components: anterior and posterior part of the default mode network, left and right frontoparietal network and salience network. No significant differences concerning fMRI measures were found across groups. However, the frontal lesions group performed significantly worse on neuropsychological tests than the other two groups. Additionally, the frontal lesions group showed a significant positive association of stronger default mode network–salience network connectivity with better executive performances. Our findings suggest that, on fMRI level, frontal network measures are not largely affected by frontal lesions following a mild to moderate traumatic brain injury. Yet, patients with damage to the frontal structures did show poorer executive abilities which might to some degree be related to altered frontal network connectivity between the default mode network and salience network.

## Introduction

Traumatic brain injury (TBI) is a major global health problem [1]. The severity of brain trauma can be classified as mild, moderate or severe based on loss of consciousness (LOC), posttraumatic amnesia (PTA) and the Glasgow Coma Scale (GCS). Mild TBI is by far the most common and comprises approximately 85–90% of all patients. Moderate TBI comprises approximately 5–10% of all patients [2]. Due to the acceleration-deceleration movement of the brain during the trauma, diffuse brain injury can also occur [3]. Moreover, areas of the brain that are especially vulnerable to the impact of a traumatic event are the frontal structures, due to the movement of the brain across various ridges and bony protuberances of the anterior cranial fossa [4]. Frontal damage on structural neuroimaging (e.g. computed tomography (CT) and magnetic resonance imaging (MRI)) is found for a small proportion of patients with mild TBI [5] and a slightly larger proportion of patients with moderate TBI [6].

In general, the presence of diffuse injury following TBI may cause a less efficient connection between functionally related brain areas [7] resulting in slower information processing speed [8]. Concerning the latter, a clear and strong positive relationship between severity of trauma and slowness of information processing speed has been established [9–11]. Furthermore, focal damage to the frontal brain structures following TBI may interrupt frontal networks [4, 12]. The frontal areas of the brain are extremely important for executive functioning, which comprises abilities to engage in everyday life situations that require monitoring, planning, flexibility and control of complex task behavior [4, 13].

Existent literature shows that executive abilities depend on flexible and effective switching between relevant networks with opposing functions [14, 15]. Better executive abilities are associated with significant negative correlations between externally oriented networks (e.g. frontoparietal/attention networks) and more internally oriented networks, like the default mode network (DMN) [15–17]. The DMN plays a crucial role in internal mental processes and is located in the ventromedial and prefrontal cortex and posterior cingulate cortex [18–20]. Executive networks, including the frontoparietal network (FPN), are crucial when it comes to flexible adaptive processes. There is substantial variation in the anatomical localization of the FPN between individuals, including the intraparietal sulcus, ventral inferior temporal lobe and localized regions of the lateral prefrontal cortex [21]. The salience network (SN) is essential for effective switching between the opposing networks and has been associated with detection of external stimuli, monitoring and cognitive control processes, and has key nodes in the anterior cingulate cortex and the anterior insular cortices [22–28]. Presumably, complaints following TBI may partly be explained by alterations in functional brain network activity and connectivity [29–31].

Considering that frontal lesions are the most prevailing focal lesions and are important for executive abilities in daily life, it is important to investigate the specific effects of frontal lesions on brain networks in brain trauma patients. Independent components analysis (ICA) commonly is used to detect distinct networks involved in various mental processes. It is assumed that frontal components that are functionally coupled at rest, are also co-activated during active cognitive processes [32].

The main objective of the present study was to investigate the impact of macro-structural lesions on frontal intrinsic network measures (resting-state fMRI) and the effect on neuropsychological measures (information processing speed and executive functions) in patients with mild to moderate TBI in the early chronic phase post-injury. Results were compared with mild TBI patients without frontal lesions and healthy controls.

## Materials and methods

### Study design and participants

This fMRI study was part of a larger prospective follow-up study on recovery after mild TBI (UPFRONT study) [33]. Data were obtained between March 2013 and December 2017. All patients were admitted to the Emergency Department of the University Medical Center Groningen (UMCG), the Netherlands, a level 1 trauma center. Mild TBI was defined by an admission Glasgow Coma Score (GCS) of 13–15, loss of consciousness ≤ 30 minutes and posttraumatic amnesia < 24 hours. Moderate TBI was defined by a GCS-score of 9–12 [34, 35].

For the present study, we included patients with mild or moderate TBI with frontal lesions on admission CT-scans (CT+ group). Additionally, we included patients with mild TBI without lesions on admission CT-scans (CT- group) and a group of healthy controls (HC group). Patients were scanned at 3 months post-injury with a 3.0 T MRI scanner and concurrently completed the neuropsychological assessment on the same day. Exclusion criteria for patients and healthy controls were: age < 18 years, history of drug or alcohol abuse, major psychiatric or neurological disorders, no permanent home address, insufficient comprehension of the Dutch language and contraindications for MRI (implanted ferromagnetic devices or objects, pregnancy or claustrophobia). Additionally, for healthy controls, previous traumatic brain injury was an exclusion criteria. Data were obtained in compliance with the ethical regulations of the UMCG. All participants gave written informed consent and procedures were executed according to the declaration of Helsinki.

### Image acquisition

A 3.0 T Philips Achieva MRI scanner (Phillips Medical Systems, Best, The Netherlands) equipped with a 32-channel SENSE head coil was used for image acquisition. A high resolution transversal T1-weighted sequence image was made for anatomical reference (TR 9 ms; TE 3.5ms; flip angle 8°; FOV 256x232x170 mm; reconstructed voxel size 1x1x1 mm). For resting-state imaging, three-hundred volumes were acquired with slices aligned in the anterior commissure (AC)-posterior commissure (PC) plane and recorded in descending order (TR 2000 ms; TE 20 ms; FOV 224x224x136.5 mm; reconstructed voxel size 3.5x3.5x3.5 mm). Participants were instructed to close their eyes and stay awake.

### Neuropsychological measures

#### Speed of information processing

The Trail Making Test (TMT) [36] consists of two parts. TMT part A is a measure for attention and information processing speed. In TMT part A, participants have to connect circled numbers as quick as possible in ascending order (1-2-3-…). Time to finish was noted and named TMTA, with lower scores reflecting better performance.

#### Frontal executive functioning

TMT part B measures mental flexibility, an aspect of executive functioning [37]. In TMT part B, participants have to connect circled numbers and letters in alternating and ascending order (1-A-2-B-…). Time to finish was noted and named TMTB, with lower scores reflecting better performance.

The Controlled Oral Word Association Test (COWAT) [38] is a measure for executive control, an aspect of executive functioning. Participants have to finish three trials in which they have to generate as many words as possible starting with a specific letter within one minute (D-A-T) while keeping three restricting rules in mind [13]. A total score of correctly named words over the three trials was calculated and named COWAT, with higher scores reflecting better performance.

#### fMRI data processing

Statistical Parametric Mapping (SPM12 Wellcome Department, University College London, London, England) implemented in Matlab (version R2014a; MathWorks, Natick, MA, USA) was used for pre-processing, which consisted of slice timing correction, image realignment to the first functional image, co-registration of functional data with individual participants’ T1-weighted images, normalization using diffeomorphic nonlinear registration tool (DARTEL) (isotropic voxels of 3x3x3 mm) to the MNI template and smoothing (8 mm full-width at half maximum (FWHM) Gaussian kernel).

Group ICA of fMRI Toolbox (GIFT) version 4.0a, implemented in Matlab, was used for spatial independent component analysis (ICA) [39]. The mean number of independent components was estimated using Minimum Description Length (MDL) and Akaike’s Information Criterion [40]. Following subject-specific PCA, group ICA was performed with the estimated number of components and ICASSO was repeated 20 times to establish the stability of component decomposition [41]. Back-reconstruction was done using spatial-temporal regression and results were scaled to the original data (i.e. % signal change). Neural components were identified visually (based on previously published literature) by H.J.v.d.H. and E.J.L. independently and by spatial regression of network templates provided with GIFT. Components corresponding with the DMN, EN and SN were selected for further analyses.

### Statistical analysis

The statistical analyses on behavioral data were performed in Statistical Package and Service Solutions (SPSS; version 23.0; Armonk, NY: IBM Corp). Assumptions were checked. Differences in demographic and clinical variables between the CT+, CT- and HC groups were examined using chi-square tests for categorical data and Mann-Whitney U tests for ordinal and nonparametric data. Differences in neuropsychological measures (TMTA, TMTB and COWAT) across groups were assessed using ANOVA with post hoc t-tests.

For the fMRI data analysis concerning component activity, first, we tested in SPM whether there was a significant difference across groups in the spatial maps of the selected components using an ANOVA (p< 0.001, FDR cluster correction p < 0.01, k > 10). Secondly, activation of the spatial maps was voxel wise related to frontal executive measures (TMTB and COWAT). Thirdly, by modelling an interaction between regressors of neuropsychological performance per group, we investigated whether there was a significant difference across groups in the relation between components activity and executive measures.

Concerning component connectivity, first, we investigated connectivity strength between components using the time courses of the selected components. Subjects’ between-component correlations were extracted from the correlation matrix of post-processed time courses created by GIFT and imported in SPSS. Secondly, ANOVA analyses were conducted to compare the Z-scores across groups. Thirdly, by modelling an interaction between regressors of neuropsychological performance per group, we investigated whether there was a significant difference across groups in the relation between components connectivity and executive measures. Finally, to investigate whether correlations between groups significantly differed, we transformed the correlations to Fisher Z scores and calculated the standard error of the difference of the Z-scores and the associated ratio of the difference to the standard error which was subsequently compared to the normal distribution. The alpha level was set at .05 for all analyses.

## Results

### Participants

We included 17 mild to moderate TBI patients with frontal lesions (CT+ group), 30 mild TBI patients without frontal lesions (CT- group) and 20 healthy controls (HC group). Table 1 presents the participant characteristics, showing that groups were well matched for age, educational level, GCS-score and handedness. However, the CT+ group consisted of significantly fewer mild TBI patients and more males compared to the CT- group.

**Table 1:** Participant characteristic, M (±SD), n (%).

	*TBI patients*		*HCs*						
	1) CT+	2) CT-	3) HCs	Difference 1–2		Difference 1–3		Difference 2–3	
Variable	(*n* = 17)	(*n* = 30)	(*n* = 20)	χ^2^/U	*p*	χ^2^/U	*p*	χ^2^/U	*p*
Age	39.7 (15.9)	36.7 (13.7)	36.2 (14.0)	U = 232.5	.618	U = 150.5	.552	U = 293	.890
Gender, male (%)	14 (82%)	16 (53%)	14 (70%)	χ^2^ = 4.0	< .05	χ^2^ = 0.8	.383	χ^2^ = 1.4	.239
Education	5.7 (0.9)	5.7 (0.9)	6.0 (0.8)	U = 245.5	.824	U = 136.5	.277	U = 253.5	.328
GCS score	13.4 (2.1)	14.3 (0.7)		U = 211	.289				
Mild TBI, %	65	100		χ2 = 12.5	< .01				
Handedness, right (%)	17 (100%)	26 (87%)	17 (85%)	χ^2^ = 2.5	.290	χ^2^ = 2.8	.096	χ^2^ = .9	.631

*Note*. CT+: mild to moderate traumatic brain injury patients with frontal lesions; CT-: mild traumatic brain injury patients without frontal lesions; HCs: healthy controls; Education = 7-point scale ranging from 1 (primary school education only) to 7 (university education); GCS: Glasgow Coma Scale; SD = standard deviation.

Information about the clinical characteristics of the CT-abnormalities are presented in S1 Table. From the entire CT+ group (n = 17), there were six mild TBI patients with a PTA < 1 hour, five mild TBI patients with PTA > 1 hour and six moderate TBI patients with PTA duration ranging from 1 hour up to 4 days (specifically: 1–24 hours (n = 1); 2 days (n = 2); 3 days (n = 1); 4 days(n = 2)).

### Neuropsychological measures

For all measures, there were no significant differences between CT- patients and healthy controls.

### Information processing speed

Across groups, the neuropsychological measure for information processing speed significantly differed (F = 3.21, p < .05). Table 2 shows that CT+ patients performed significantly worse than the CT- patients on the TMTA.

**Table 2:** Comparisons of scores on tests for information processing speed and executive functions across groups M (±SD).

	*TBI patients*		*HCs*						
	1) CT+	2) CT-	3) HC	Difference 1–2		Difference 1–3		Difference 2–3	
Variable	(*n* = 17)	(*n* = 30)	(*n* = 20)	t	*p*	t	*p*	T	*p*
Information processing speed									
TMT A	34.2 (15.0)	25.6 (11.8)	26.6 (7.7)	2.19	< .05	-1.91	.069	.33	.744
Frontal executive functions									
TMT B	76.1 (35.0)	51.4 (13.8)	59.6 (21.4)	-2.71	< .05	1.71	.096	1.60	.115
COWAT	33.3 (13.1)	38.8 (12.8)	41.9 (11.2)	1.37	.177	-2.13	< .05	.87	.390

*Note*. TBI patients were tested at 3 months post-injury; CT+: mild to moderate traumatic brain injury patients with frontal lesions; CT-: mild traumatic brain injury patients without frontal lesions; HCs: healthy controls; TMT A: Trail Making Test A; TMT B: Trail Making Test B; COWAT: Controlled Oral Word Association Test; SD = standard deviation.

### Frontal executive functions

Across groups, the frontal executive measure for mental flexibility (TMTB) significantly differed (F = 5.88, p < .01). Table 2 shows that CT+ patients performed significantly worse than the CT- patients on the TMTB.

The frontal executive measure for executive control (COWAT) did not significantly differ across groups (F = 2.19, p = 0.121). Yet, Table 2 shows that CT+ patients performed significantly worse on the COWAT than healthy controls.

### Components

**Fig 1:**
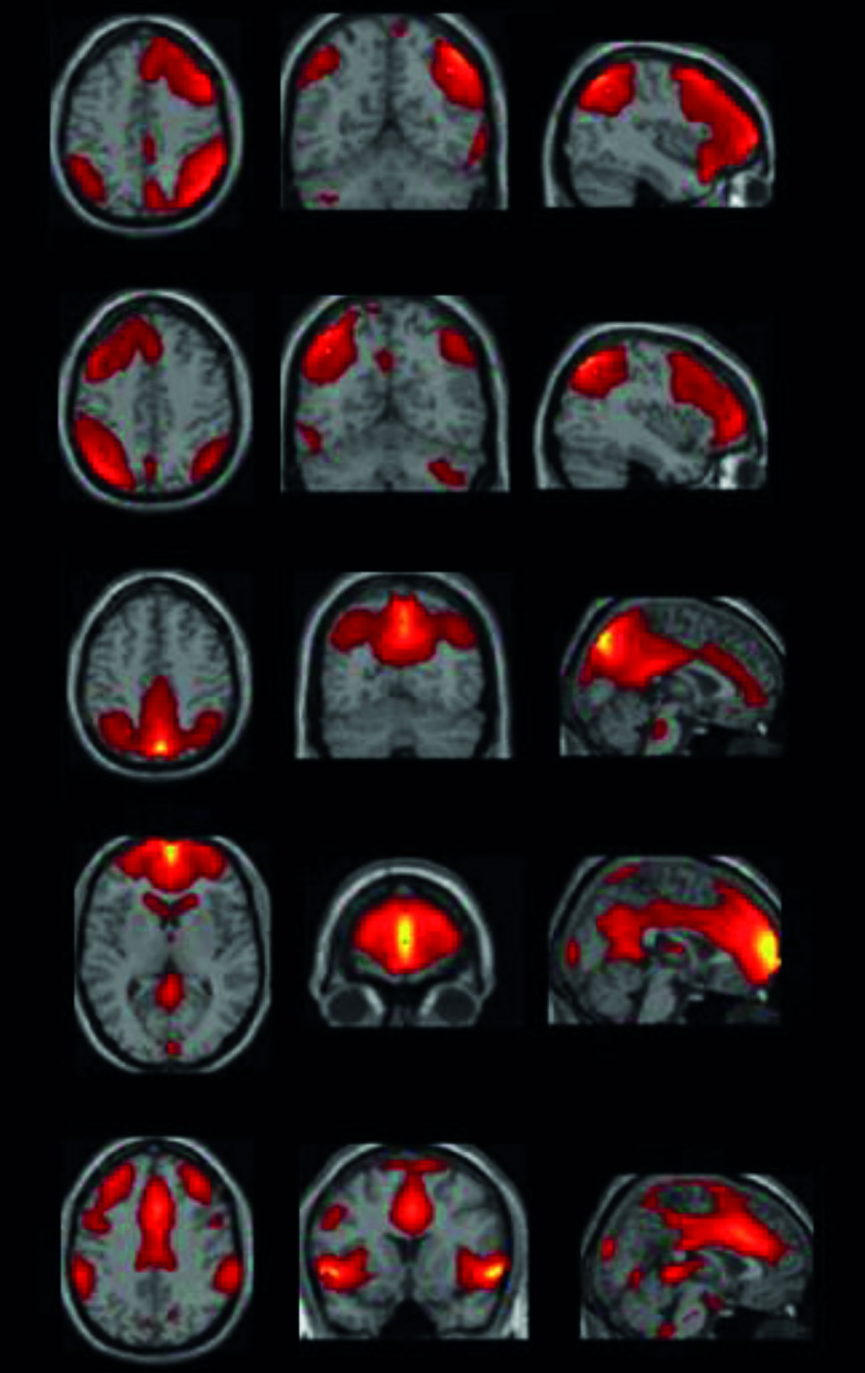
From top to bottom: right frontoparietal network, left frontoparietal network, posterior default mode network, anterior default mode network and salience network.

ICA resulted in 29 components from which we selected six components of interest. Components included were the anterior and posterior part of the default mode network (DMN), the left and right frontoparietal network (FPN), dorsal attention network (DAN) and salience network (SN). For an overview, see Fig 1. Since in the present study we focused on frontal functioning, the DAN component was not included in further analyses.

### Activity of frontal components and relations with frontal executive measures

Across the CT+, CT- and HC groups, no significant differences were found regarding spatial maps of the components, indicating no significant differences in component activity. Furthermore, spatial maps of these frontal components were not significantly correlated with the frontal executive measures (TMTB and COWAT), nor did these relations between spatial maps and the frontal executive measures significantly differ across the groups.

### Connectivity between frontal components

Table 3 shows that there were no significant differences across the CT+, CT- and HC groups concerning the correlation strength between the frontal components.

**Table 3:** Comparisons of connectivity between frontal components across groups, mean Z-scores (SD).

	*TBI patients*		*HCs*		
	1) CT+	2) CT-	3) HCs	Difference 1-2-3	
	(*n* = 17)	(*n* = 30)	(*n* = 20)	F	*P*
right FPN ‐ left FPN	.29 (.26)	.34 (.58)	.44 (.48)	.46	.631
right FPN–posterior DMN	22 (.21)	.32 (.50)	.20 (.21)	.76	.470
right FPN–anterior DMN	14 (.15)	.07 (.21)	.13 (.22)	.94	.395
right FPN–SN	.30 (.23)	.28 (.36)	.23 (.30)	.23	.798
left FPN ‐ posterior DMN	.05 (.25)	.05 (.36)	.17 (.21)	1.08	.347
left FPN–anterior DMN	.24 (.34)	,09 (.23)	,13 (.29)	1.40	.253
left FPN ‐ SN	-.05(.32)	-.10 (.37)	-.08 (.22)	.14	.869
posterior DMN–anterior DMN	.44 (.33)	.40 (.25)	.40 (.26)	.17	.845
posterior DMN ‐ SN	-.17 (.23)	-.03 (.53)	-.11 (.18)	.73	.485
anterior DMN ‐ SN	-.13 (.39)	-.27 (.41)	-.18 (.19)	.94	.394

*Note*. CT+: mild to moderate traumatic brain injury patients with frontal lesions; CT-: mild traumatic brain injury patients without frontal lesions; HCs: healthy controls; FPN: frontoparietal network; DMN: default mode network; SN: salience network.

### Connectivity between frontal components with frontal executive measures

Table 4 shows the correlation strength between the frontal components with the frontal executive measures (TMTB and COWAT) for each group independently. Only the CT+ group showed a significant positive association of stronger DMN–SN connectivity with better executive performances. Specifically, a stronger posterior DMN ‐ SN connectivity was significantly associated with better mental flexibility (TMTB). Additionally, a stronger anterior DMN ‐ SN connectivity was significantly associated with better executive control (COWAT). For the CT- group and HC group, opposite significant moderate to strong associations were found of weaker components (FPN, DMN, SN) connectivity is significantly associated with better executive control (COWAT). When comparing these correlations across groups, we found that in the CT+ group, the stronger anterior DMN—SN connectivity that was significantly associated with better executive control also significantly differed from the other two groups (p = < .01).

**Table 4:** Correlations between component connectivity and executive performances.

	Patients				HCs	
Variables	CT+		CT-		HCs	
	(n = 17)		(n = 31)		(n = 20)	
	TMTB	COWAT	TMTB	COWAT	TMTB	COWAT
right FPN–post. DMN	-.40	-.38	-.28	.28	-.15	-.23
right FPN–ant. DMN	-.02	.15	.21	-.45*	.14	-.34
right FPN–SN	-.40	.45	-.17	.14	.23	-.05
left FPN–post. DMN	.43	-.32	-.18	.21	-.29	-.26
left FPN–ant. DMN	.41	-.02	.06	-.10	.29	-.58**
left FPN ‐ SN	-.06	.31	.22	-.38*	.11	-.09
post. DMN ‐ SN	-.50*	.47	-.23	.07	.00	-.28
ant. DMN ‐ SN	-.31	.51*	.30	-.43*	.26	.02

*Note*. CT+: mild to moderate traumatic brain injury patients with frontal lesions; CT-: mild traumatic brain injury patients without frontal lesions; HCs: healthy controls; TMT B: Trail Making Test B; COWAT: Controlled Oral Word Association Test; FPN: frontoparietal network; DMN: default mode network; SN: salience network.

* p< .05

** p < .01

## Discussion

The present study investigated whether frontal lesions following mild to moderate TBI would negatively affect intrinsic network measures by examining frontal network activity and connectivity and frontal executive measures. When solely looking at the fMRI measures of the brain networks, no apparent differences across the groups could be detected. However, patients with frontal lesions showed significantly poorer executive abilities when compared to the control groups (mild TBI patients without frontal lesions and healthy controls). The control groups did not significantly differ from each other. Remarkably, only for the frontal lesions group, stronger resting-state connectivity between the DMN and SN appeared related to better executive performances.

The finding that patients with frontal lesions exhibited poorer frontal executive functions regarding mental flexibility and executive control is in line with our expectation that damage to frontal structures as noted on the CT-scans can compromise executive functioning. Furthermore, we found that the frontal lesions group also exhibited a slower information processing speed in comparison to the patient group without CT-abnormalities. Presumably, this is a more subtle consequence of the fact that the frontal lesions group is on average more severely injured, even though, based on the PTA duration, we included patients at the rather mild end of the PTA spectrum and the GCS-scores did not significantly differ.

When considering the fMRI measures of the frontal components, we found no evidence that either component activity or connectivity between frontal components significantly differed across groups. Apparently, the present methodology does not detect evident alterations as shown by frontal CT-abnormalities on frontal network functioning on fMRI level. Yet, when correlating the resting-state connectivity between frontal components with frontal executive measures, it is striking that only for the frontal lesions group, a stronger DMN ‐ SN connectivity was found to be related to better frontal executive performances.

The finding that stronger connectivity between the DMN and SN is associated with better executive abilities may indicate altered functional network connectivity in this group. Another argument for this assumption is that this stronger anterior DMN ‐ SN connectivity significantly differed from other non-frontal groups. Based on prior research, we expected an opposed relationship with weaker connectivity between the DMN and the executive/attention networks, reflecting efficient switching between the brain networks, in relation to better executive abilities [14, 15]. This is also highlighted by the present findings in the non-frontal groups. Possibly, the stronger co-activation of the DMN and SN in the frontal lesions group is an attempt to compensate for the frontal damage (that may cause the connection between the DMN and SN to be less efficient) to still keep performing at a premorbid level of executive functioning. Yet, this still is not quite as effective considering that the frontal lesion patients performed significantly worse on the executive tests. Previous research by Bonelle and colleagues also demonstrated that the SN plays a crucial role in the regulation of DMN activity and that failure of deactivation of the DMN is related to impaired cognitive control [22]. This is also in line with other existent literature, reporting stronger resting-state connectivity between the DMN and executive networks following brain trauma [29–31].

The present study is subject to several limitations. The available data allow us to investigate several differences and correlations but we are unfortunately unable to make any causal inferences. Furthermore, CT-scans have a limited sensitivity when compared to for example MRI-scans, which may also in part explain the lack of significant differences between groups. Furthermore, although no differences were present regarding GCS scores between the frontal and nonfrontal groups, the duration of PTA was slightly higher and therefore the CT group should be regarded as representing the more severe end of the mild TBI spectrum. Also, the poorer information processing speed found in the frontal lesions group may in part have negatively affected the executive measures. Finally, other networks or brain areas may be involved in changes caused by the lesions that are not included in the present study.

## Conclusions

To conclude, present findings indicate that frontal lesions following mild to moderate TBI do not evidently affect frontal network functions on a fMRI level. However, patients with frontal lesions did show poorer executive abilities on neuropsychological tests that may partly be related to an imbalance in connectivity between the DMN and SN. This finding deserves further exploration in future studies.

## Supporting information

S1 Table Characteristics of CT-lesions. (DOCX)
