# Supplementary material for: The impact of frontal lesions after mild to moderate traumatic brain injury on frontal network measures
Source: PLoS One. 2023 Nov 30;18(11):e0287832. doi: 10.1371/journal.pone.0287832 (PMC10688722; doi:10.1371/journal.pone.0287832)
Supplement: S1 Table — (DOCX) [file pone.0287832.s001.docx]

| Supplementary Table 1 | | | | | |
| --- | --- | --- | --- | --- | --- |
| *Characteristics of CT-lesions* | | | | | |
| Patient | Marshall Score | Midline Shift | Contusion (volume in cm3) | EDS/SAH/SDH | Micro-hemorrhages |
| 1 | 2 | no | frontal, left (0.1) | SAH |  |
| 2 | 2 | no | frontal, left (0.34) | SAH | cortical |
| 3 | 5 | 6 mm | frontal, right (60) | EDH |  |
| 4 | 2 | no | frontal, left & right (2 & 4) | EDH |  |
| 5 | 2 | no |  | EDH |  |
| 6 | 2 | no | frontal, left (0.05) |  |  |
| 7 | 2 | no | frontal, right (0.11) | SAH |  |
| 8 | 2 | no | frontal, right (0.9) | SDH |  |
| 9 | 2 | no | frontal, left (20.1) |  |  |
| 10 | 2 | no | frontal, left (0.19) |  | cortical |
| 11 | 2 | no | frontal, left & right (8.4 & 12.6) | SAH |  |
| 12 | 2 | no |  | SDH/SAH |  |
| 13 | 2 | no | frontal, left>right (11.3) | SDH/EDH/SAH |  |
| 14 | 2 | no | frontal, right (3.1) | EDH/SDH |  |
| 15 | 5 | 6mm | frontal, right (1.5) | EDH/SDH/SAH | cortical, subcortical |
| 16 | 2 | no |  | SDH, SAH |  |
| 17 | 2 | no |  |  | cortical, subcortical |
| *Abbreviations:* EDH = epidural hematoma, SAH = subarachnoid hemorrhage, SDH = subdural hematoma. | | | | | |
